# Childhood traumatic experiences and postpartum depression: a mixed methods narrative study

**DOI:** 10.1007/s00737-026-01777-0

**Published:** 2026-10-17

**Authors:** Maria McDonald, Richard Jiang, Sahana Gopi, Jennifer L. Payne, Xin Tong

**Affiliations:** University of Virginia, Charlottesville, USA

**Keywords:** Postpartum depression, Childhood traumatic events scale, Topic modeling, Thematic analysis, Sentiment analysis

## Abstract

**Purpose:**

Postpartum depression (PPD) is a significant public health concern with profound effects on the health and well-being of women, children, and families. A growing body of research has identified an association between childhood traumatic experiences and an increased risk of PPD. This study aims to elucidate potentially underrecognized childhood experiences that are perceived as traumatic or particularly formative, and to examine whether linguistic features in brief trauma narratives are associated with PPD symptomatology.

**Methods:**

This analysis included data from 143 participants who provided narrative responses to an open-ended item on the Childhood Traumatic Events Scale (CTES). Topic modeling and thematic analysis were used to identify recurring trauma-related themes, while dictionary-based sentiment analysis quantified the emotional valence of participants’ responses. Pearson’s correlation analyses examined associations between sentiment scores and PPD severity across four postpartum time points (Week 2, Week 6, Month 3, and Month 6).

**Results:**

Three primary themes emerged: (1) familial adversity, (2) verbal/emotional abuse, and (3) childhood relocation or moving. Across all narratives, sentiment scores indicated predominantly negative language. No significant associations were found between sentiment scores and PPD symptomatology through six months postpartum.

**Conclusion:**

Expanding assessment tools to include open-ended narrative prompts may strengthen the identification and characterization of childhood adversity in both research and clinical settings. Further research is needed to better understand whether and how narrative language reflects perinatal mental health.

**Clinical trial number:**

Not applicable.

**Supplementary Information:**

The online version contains supplementary material available at https://doi.org/10.1007/s00737-026-01777-0.

## Highlights

This analysis examined childhood trauma narratives from 143 perinatal women who completed the Childhood Traumatic Events Scale.

Three primary themes emerged: familial adversity, verbal/emotional abuse, and childhood relocation.

Narratives were characterized by predominantly negative sentiment (or emotional valence).

Narrative sentiment was not associated with postpartum depression severity across the first six months postpartum.

**Supplementary Information:**

The online version contains supplementary material available at https://doi.org/10.1007/s00737-026-01777-0.

## Introduction

Postpartum depression (PPD) is a prevalent and serious public health concern, affecting approximately 13% of postpartum women in the United States and contributing to adverse outcomes in maternal functioning, mother-infant bonding, and children’s cognitive, emotional, and social development (Bauman et al. 2020; Darcy et al. 2011; Feldman et al. 2009; Simonovich et al. 2021). Emerging evidence suggests that childhood adversity is an important contributor to PPD vulnerability (Fu et al. 2024; Racine et al. 2021). This association is thought to reflect both psychosocial mechanisms, such as trauma-related cognitive and relational challenges, and persistent alterations in biological systems, including dysregulation of the stress-response system and neuroendocrine functioning (Berens et al. 2017; Lev-Wiesel et al. 2009; Major Depressive Disorder Working Group of the Psychiatric Genomics Consortium et al. 2018; Teicher et al. 2016). In recognition of these risks, the American College of Obstetricians and Gynecologists (ACOG) recommends routine assessment of lifetime trauma exposure and the integration of trauma-informed care into perinatal practice (ACOG, 2021).

Assessment tools, such as the Adverse Childhood Experiences Questionnaire (ACE-Q) and the Childhood Trauma Questionnaire (CTQ), are widely used to evaluate exposure to childhood adversity, including abuse, neglect, and household dysfunction, and have demonstrated associations with adverse perinatal outcomes, including pregnancy complications and adverse birth outcomes (Mamun et al. 2023). However, reliance on predefined categories of adversity may limit the ability to capture the full range of potentially traumatic experiences with clinical relevance (Lacey and Minnis 2020; Mendel et al. 2021). Instead, the Childhood Traumatic Events Scale (CTES) offers a complementary approach by assessing five core domains of childhood trauma while incorporating an open-ended item that allows respondents to describe additional events they perceive as particularly traumatic or formative (see Supplemental Table 1) (Pennebaker and Susman 1988).

Narrative responses to the CTES have not been systematically examined in prior research and may provide clinically meaningful insight into trauma exposures not captured by commonly used assessment tools (McDonald et al. 2026). In parallel, advances in text analytic methods enable the evaluation of linguistic features, including sentiment (i.e., emotional valence), as potential indicators of psychological functioning. Prior work, informed by Freud’s theory of parapraxes, or “slips of the tongue,” suggests that linguistic patterns in written and spoken language, including more negatively valenced language, can indicate depressive symptomatology; however, this framework has received limited empirical attention in the context of PPD (Landoni et al. 2023; Pennebaker et al. 2003; Ramirez-Esparza et al. 2008).

Using a mixed-methods design, this study analyzes narrative responses to the CTES in a perinatal sample to identify potentially underrecognized trauma exposures. A secondary aim is to assess whether linguistic features (i.e., emotional valence) in brief narratives can serve as potential indicators of PPD symptomatology. By integrating respondent-reported accounts of childhood trauma with computational text analysis, this study aims to advance a more comprehensive and clinically relevant understanding of childhood adversity and PPD risk.

## Materials and methods

### Study design

This study is a secondary analysis of data from 143 participants enrolled in a multi-site, prospective cohort study conducted between November 2014 and January 2024 to identify biomarkers and psychosocial risk factors for PPD. The parent prospective study included 506 participants, of whom 143 provided a narrative response to the CTES. Participants in the parent prospective study completed baseline surveys during the first or second trimester of pregnancy, which included sociodemographic questionnaires and the CTES. PPD symptoms were assessed using the Edinburgh Postnatal Depression Scale (EPDS) at four postpartum time points: Week 2 (W2), Week 6 (W6), Month 3 (M3), and Month 6 (M6). Eligible participants were pregnant women aged 18 years or older with a healthy pregnancy and less than 24 weeks gestation at enrollment. Exclusion criteria included not being pregnant at initial screening, being younger than 18 years, or having an unstable medical condition (i.e., current substance use or depression with active suicidal ideation). Participants were recruited from (redacted for review) and (redacted for review) through print and online research listings, in-person outreach, and advertisements in public areas on university campuses.

### Measures

#### Childhood traumatic events scale (CTES)

The CTES consists of six items that assess exposure to potentially traumatic experiences before the age of 17. Each item includes a binary response to indicate exposure, denoted as “[0] no” or “[1] yes”. This study examined narrative responses to item six on the CTES, which specifically asks, *“Prior to the age of 17*,* did you experience any other major upheaval that you think may have shaped your life or personality significantly?”* Participants who responded “[1] yes” to this question were prompted to describe the event in a follow-up question

#### Edinburgh postnatal depression scale (epds)

The EPDS is a widely used 10-item questionnaire designed to screen for symptoms of prenatal and postpartum depression. Translated into more than 50 languages, it is the most commonly used tool for assessing depressive symptoms in the perinatal period (Cox et al. 1987). Each item on the EPDS is rated on a 4-point Likert scale (0–3), yielding a total score of up to 30. Higher scores indicate more severe symptoms, with scores greater than 13 demonstrating strong correlation with diagnostic criteria for major depressive episodes as defined in the DSM-5 (Levis et al. 2020). This study examined EPDS scores as a continuous variable reflecting PPD symptom severity at each postpartum timepoint.

### Data analysis

#### Topic modeling and thematic analysis

Topic modeling was conducted by two authors (initials redacted) to identify topics frequently reported and their respective keywords. Latent Dirichlet Allocation (LDA) was applied using the *topicmodels* package in R to evaluate word frequency and the conditional probability of individual words being associated with certain topics. This approach is intended to identify the most salient terms identifying a topic, which facilitates interpretation and topic labeling (Blei et al. 2003). Each open-ended response was treated as a distinct document, yielding a total of 143 unique documents. Aligned with valid and reliable data-generating processes, the number of topics represented in the documents were determined based on fit statistics and author consensus (Maier et al. 2018).

For additional human validation and enhanced reliability, two co-authors who were not involved in the topic modeling analysis (initials redacted) manually coded all documents to identify emerging themes (O’Connor and Joffe 2020). Following Braun and Clarke’s (2006) six-stage thematic analysis process, the authors generated initial codes and identified recurring themes across documents. All team members discussed the outcomes of topic modeling and thematic analysis to reach final consensus on the most frequently occurring trauma-related topics.

#### Sentiment analysis

Following topic modeling and thematic analysis, sentiment analysis was conducted using a lexicon-based approach. The aim of a sentiment analysis is to extract meaningful information from text using natural language processing to determine the responder’s emotion or attitude, which can be categorized as positive, negative, or neutral (Nandwani and Verma 2021). Most often, sentiment analysis is applied to elucidate the polarity or valence of a document, indicating how positive or negative it is overall (Cero et al. 2024). Compared with machine learning approaches, lexicon-based approaches are often used given their practicality and reliability, particularly in smaller datasets (Khoo and Johnkhan 2018).

This study employed the AFINN, Bing, and NRC lexicons, which consist of English words, are publicly available, and are among the most popular lexicons applied in sentiment analysis research (Cero et al. 2024). A list of example words and sentiment values from the three lexicons was generated using the *textdata* package in R and is outlined in Supplemental Table 2. While lexicon-based sentiment analysis has been widely applied in research, limitations regarding its validity for short texts have been reported in the prior literature (Boyd 2017; Hunter and Grant 2025). Thus, sentiment scores were compared across these three lexicons to generate a more robust evaluation of emotional assessments and their associations with PPD outcomes.

The AFINN dictionary contains 2,477 words scored on a scale from − 5 to + 5, where negative scores (< 0) indicate negative sentiment, 0 indicates neutral sentiment, and positive scores (> 0) indicate positive sentiment (Bradley and Lang 1999; Nielsen 2011). The Bing dictionary includes 6,786 words and uses a simpler classification, labeling each word as either positive or negative (Hu and Liu 2004). The NRC dictionary contains the largest set of words (13,872) and links each word to one or more primary emotions (i.e., anger, anticipation, fear, surprise, sadness, joy, disgust, and trust) (Mohammad and Turney 2013).

To estimate sentiment scores for each document, an algorithm aggregated word-level sentiment scores to produce a document-level sentiment score (Khoo and Johnkhan 2018). Consistent with established sentiment analysis guidelines, stop words (i.e., common function words such as “and” and “the”) were first removed, as they are considered uninformative and do not convey sentiment (Cero et al. 2024). Using the *tidytext* R package, the remaining words were scored according to each lexicon and aggregated to generate an average sentiment score for each document (Cero et al. 2024; Demiroz et al. 2012). For the AFINN lexicon, document-level sentiment was calculated by summing the sentiment scores of all matched words and dividing by the total number of scored words. For the Bing lexicon, positive and negative words were converted to numerical values (+ 1 for positive and − 1 for negative), summed within each document, and divided by the total number of scored words. For the NRC lexicon, words associated with anger, fear, disgust, and sadness were assigned a value of − 1, whereas words associated with anticipation, surprise, joy, and trust were assigned a value of + 1. These values were summed and divided by the total number of scored words to obtain the document-level average. Consistent with prior literature, documents with negative scores were classified as negatively valenced, and those with positive scores were classified as positively valenced (Mamo et al. 2024).

#### Correlation analysis

In the final analysis, we examined whether the average sentiment score in each document was associated with PPD symptomatology at four postpartum time points (W2, W6, M3, and M6). Pearson’s correlation analysis was used to assess the relationship between document-level sentiment scores and EPDS scores at each time point, using a significance level of 0.05.

## Results

### Participant demographics

A total of 143 participants responded “[1] yes” to item six on the CTES and completed the follow-up prompt describing their childhood experience. There were no missing narrative responses among those who endorsed this item. Full participant demographic characteristics are presented in Table 1. The mean maternal age was 33.1 years. Most participants identified as non-Hispanic White (*n* = 110, 76.9%), were married (*n* = 114, 79.7%), and held a bachelor’s degree or higher (*n* = 116, 81.1%). The number of participants who completed the EPDS at each time point, along with mean scores, were as follows: W2: *n* = 96, M = 6.2; W6: *n* = 95, M = 6.4; M3: *n* = 92, M = 5.9; and M6: *n* = 75, M = 6.0. Compared with participants in the larger multi-site prospective study who responded “[0] no” to item six and did not provide a narrative response (*n* = 363), participants in this study (*n* = 143) did not differ significantly in maternal age, race/ethnicity, marital status, or educational attainment. However, a higher prevalence of participants who responded “[1] yes” to item six had EPDS scores exceeding the standard cutoff of 13, indicating probable PPD at least once during the postpartum period (23.1%), compared with those who responded “[0] no” (13.2%).

**Table 1:** Summary of participant characteristics

Participant Characteristics (Total: *N* = 143)	Frequency (%)
Age	
20–24	5 (3.5%)
25–34	82 (57.3%)
35+	56 (39.2%)
Race and Ethnicity	
Non-Hispanic, White	110 (76.9%)
Non-Hispanic, Black	18 (12.6%)
Asian or Pacific Islander	8 (5.6%)
Other or Multiracial	7 (4.9%)
Marital Status	
Married	114 (79.7%)
Single	11 (7.7%)
Other	18 (12.6%)
Educational Attainment	
Some High School	1 (0.7%)
High School Degree	6 (4.2%)
Some College	20 (14.0%)
Bachelor’s +	116 (81.1%)
Postpartum Depression Symptoms *(EPDS score ≥ 13)*	
No	110 (76.9%)
Yes	33 (23.1%)

### Topic modeling and thematic analysis

**Fig. 1:**
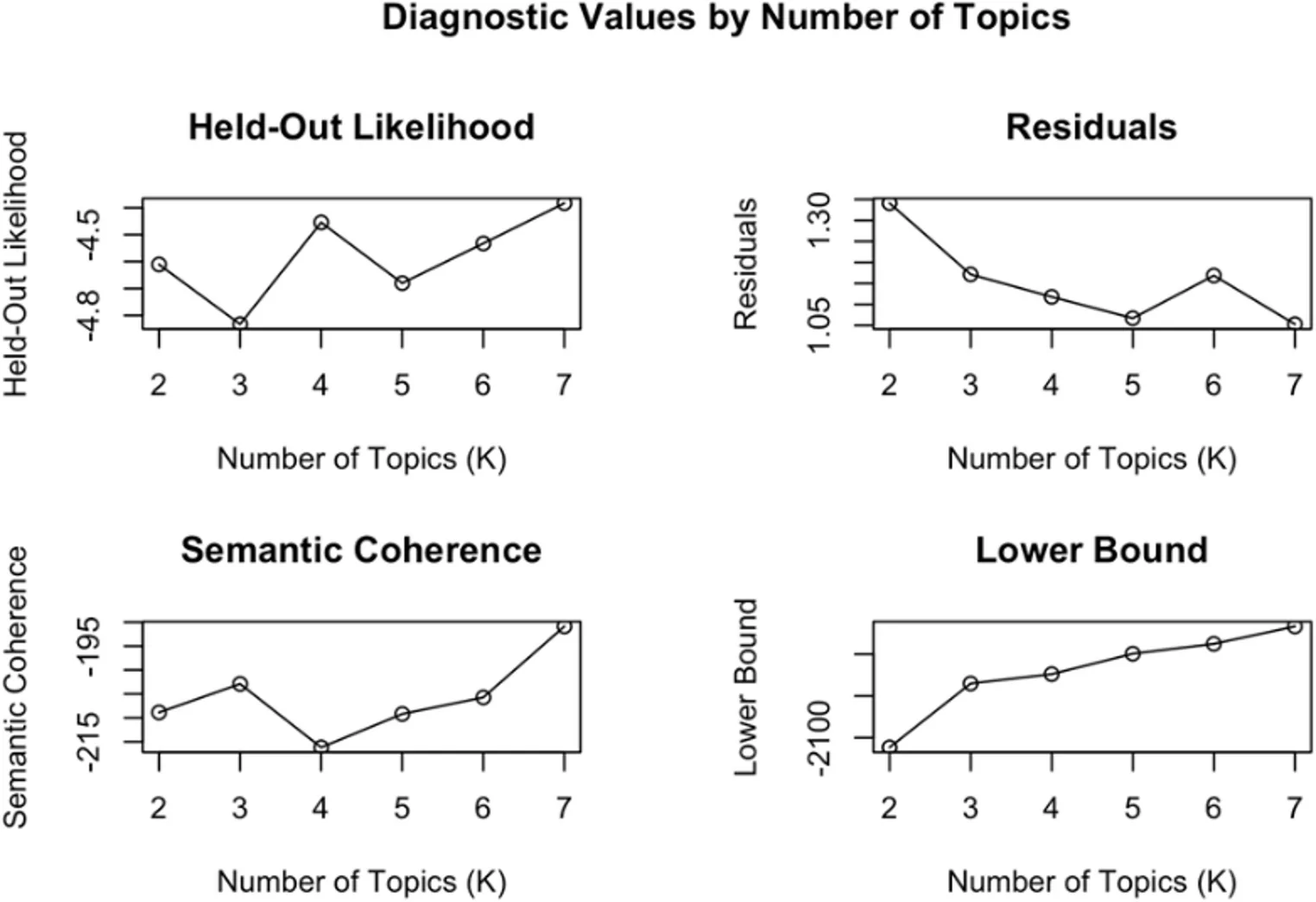
Fit statistics for topic modeling

Figure 1 displays the fit statistics for the topic modeling solutions. We identified three-, four-, and seven-topic solutions as candidate models. Among these, the three-topic model was the most parsimonious while maintaining a relatively high coherence score. The three-topic model was also the most interpretable, producing sets of keywords with clear, distinct meanings (see Table 2). Topic one prominently included words referring to family members, such as “mother,” “father,” “brother,” “step [family member],” “sister,” and “dad.” Topic two centered on abuse, particularly “emotional” and “verbal” abuse. Topic three focused on experiences related to moving during childhood.

**Table 2:** Keywords in candidate models for three, four, and seven topics

	Three-Topic Model	Four-Topic Model	Seven-Topic Model
Topic 1	Mother*** Father Brother Step Sister Dad	Abuse*** Mother*** Father Emotional Home Verbal	Parents*** Dad Family Lot Sister Job
Topic 2	Abuse*** Mother Father Emotional Parents Verbal	Mother*** Father Step Brother Sister Accident	Abuse*** Emotional Father Verbal Bullying Psychological
Topic 3	Moved*** Moving US Age Family School	Moved*** Moving US Age Family New	Mother*** Father Moving Living Life Due
Topic 4		Parents*** Suicide Dad Mom Lot Bullying	Moved*** US Age Moving New Family
Topic 5			Mom*** Sister Suicide Died Became Accident
Topic 6			Mother*** Parents Father Step Started Brother
Topic 7			Home*** School Placed Got Emotionally Care

***Indicates the word with the highest probability of belonging to the topic

Independent thematic analysis further supported the three-topic solution and provided additional context for refining topic labels. The final three topics were defined as: (1) *familial adversity*, including violence between family members (often violence toward the mother) and/or substance use within the family; (2) personal experiences of *verbal/emotional abuse*, including abuse by family members and/or peer bullying; and (3) *moving or relocation*, including international or domestic moves, school changes, transitions between parents’ homes following divorce or separation, and housing displacement. Aligned with topic 3, participants also frequently reported the number of relocations experienced and the developmental age at which these relocations occurred.

### Sentiment analysis

In the next analysis, the sentiment of participant narratives was assessed using dictionary-based sentiment analysis with the AFINN, Bing, and NRC lexicons. Across all documents, the corpus (or dataset) contained approximately 1,300 total words, of which 681 remained after removal of stop words. Table 3 presents the number of words matched to each lexicon, including the distribution of positive and negative words across documents. Overall, 110 words matched the AFINN lexicon, 99 matched the Bing lexicon, and 631 matched the NRC lexicon. Across all lexicons, participants’ language showed a notable skew toward negative sentiment, with more negatively valenced words than positively valenced words identified across documents.

**Table 3:** Matched words using the AFINN, bing, and NRC lexicon

Lexicon	Number of Matched Words	Number of Positive Words	Number of Negative Words
AFINN	110	16	94
Bing	99	9	90
NRC	631	286	345

### Correlation between sentiment scores and EPDS severity

In the final analysis, we examined Pearson’s correlation coefficients to assess the relationship between document-level sentiment scores, derived from the AFINN, Bing, and NRC lexicons, and EPDS scores at each postpartum time point. Using the AFINN and NRC lexicons, weak positive associations were observed at W2, indicating that documents exhibiting greater negativity were associated with lower EPDS scores at two weeks postpartum (AFINN: *r* =.14, *p* =.18; NRC: *r* =.11, *p* =.26). However, these associations did not reach statistical significance, based on a significance threshold of *p* <.05. No relationship between document-level sentiment scores and EPDS severity was observed using the Bing dictionary or at any other postpartum timepoint (see Supplemental Table 3).

## Discussion

Two primary findings emerged from this study. First, participants most frequently identified familial adversity, verbal/emotional abuse, and experiences of moving or relocating as particularly formative childhood events. Although the CTES does not include structured items assessing familial adversity or verbal/emotional abuse, these exposures are captured in other established instruments, such as the ACE-Q and CTQ (Bernstein et al. 2003; Felitti et al. 1998). The prominence of these themes underscores their prevalence and continued relevance for trauma screening and clinical assessment. In contrast, relocation is not assessed in most trauma-based assessment tools, despite growing evidence linking childhood relocation to adverse mental health outcomes across the lifespan (Cramm et al. 2019; Sabel et al. 2024; Valente and Vidal 2026). For example, in a study by Sabel et al. (2024), individuals who moved residential neighborhoods at least once between ages 10 and 15 had a 41% increased risk of depression in adulthood, with risk rising to 61% among those who moved two or more times. These findings may be particularly relevant for children in military families, who often experience frequent relocations and may also be affected by family separation associated with military service (Cramm et al. 2019). In this study, both the frequency of relocations and developmental age at the time of relocation emerged as salient features in topic modeling and thematic analysis, suggesting that these dimensions may influence how relocation experiences are perceived and interpreted. Collectively, these findings indicate that trauma-based screening tools and clinical assessments may benefit from explicitly evaluating relocation experiences, including their timing and contextual circumstances.

Second, despite inherent limitations, dictionary-based sentiment analysis demonstrated feasibility for characterizing the emotional tone of brief CTES narratives. This approach provided an additional layer of interpretation by quantifying both the direction (positive, negative, or neutral) and the magnitude of emotional sentiment. Overall, narratives were characterized by predominantly negatively valenced language, consistent with the prompt’s focus on “major childhood upheavals.” However, contrary to expectations, negative sentiment scores were not associated with PPD symptomatology. Instead, a trend observed in the early postpartum period suggested that more negatively valenced narratives were associated with lower EPDS scores at two weeks postpartum. This timepoint falls within the period when transient mood disturbances, commonly referred to as “baby blues”, may occur and, in some cases, progress to PPD (Tosto et al. 2023). This finding aligns, albeit modestly, with evidence that narrative disclosure of traumatic experiences may confer psychological benefits (Harrington et al. 2018; Landoni et al. 2023; Pennebaker and Susman 1988). Specifically, confiding about such experiences may facilitate psychological processing and, in turn, promote well-being in the early postpartum period. Additional longitudinal research is needed to clarify the relationship between emotional valence, the emergence and progression of PPD symptomatology, and the potential benefits of trauma disclosure.

### Strengths and limitations

This study has several notable strengths. We used a mixed-methods design that integrated multiple analytic approaches to enhance rigor. Notably, the inclusion of multiple sentiment lexicons improved the reliability and validity of sentiment estimates and their associations with PPD outcomes. Additionally, the prospective design reduced the likelihood of information bias in assessing PPD outcomes and allowed for evaluation of associations between sentiment and PPD across multiple postpartum time points.

Nevertheless, there are important limitations of this study. The sample size was relatively small, limiting the amount of text available for analysis. Although lexicon-based approaches can be effective in smaller datasets (Khoo and Johnkhan 2018), the number of matched words within each lexicon was limited. The Bing lexicon yielded the fewest matches and was less effective for assessing the severity of specific exposures. While we used widely available lexicons, larger samples are needed in future studies to generate more robust and generalizable findings. Emerging tools such as the Linguistic Inquiry and Word Count (LIWC) may also be useful in future research given their established reliability in capturing emotional and semantic features in trauma narratives (Tausczik and Pennebaker 2010).

Another limitation is the limited diversity of the sample, which was largely non-Hispanic White, married, highly educated, and reported low PPD symptoms. Several barriers can limit research participation among underrepresented populations, including language and cultural differences, stigma, limited knowledge about the health condition, prior experiences of discrimination, and not perceiving oneself as a potential candidate for research (Knapp et al. 2025). Researchers must carefully consider barriers and facilitators to research participation to reduce population exclusion and improve the generalizability of study findings. The parent multi-site prospective study excluded individuals with unstable medical conditions, including those with current substance use and depression with active suicidality. Because these populations are more likely to have experienced childhood adversity, our findings may also have limited generalizability to individuals at elevated risk for both trauma exposure and severe PPD (Broekhof et al. 2023; Bruffaerts et al. 2010).

Importantly, research indicates that individuals from racially and ethnically minoritized groups are more likely to experience socioeconomic adversity in childhood, including economic hardship, parental separation, and familial incarceration, all of which are linked to poorer long-term health outcomes (Zhang and Monnat 2022). These populations may also encounter stressors not typically captured in standard trauma assessments, such as racism and discrimination. Efforts to expand assessment tools to include such exposures represent an important step toward more inclusive and comprehensive evaluation (Bernard et al. 2021). In addition, incorporating open-ended narrative prompts can provide a richer understanding of experiences perceived as particularly traumatic or formative. This study highlights the value of such approaches in capturing a broader range of adverse exposures and their potential impact on perinatal outcomes.

## Supplementary Information

Below is the link to the electronic supplementary material.

Supplementary Material 1 (PDF 75.1 KB)

## Data Availability

The data supporting this study are available upon request.
