# Supplementary material for: Childhood traumatic experiences and postpartum depression: a mixed methods narrative study
Source: Arch Womens Ment Health. 2026 Oct 17;29(6):141. doi: 10.1007/s00737-026-01777-0 (PMC13676496; doi:10.1007/s00737-026-01777-0)
Supplement: Supplementary file 1 [file 737_2026_1777_MOESM1_ESM.pdf]

Supplemental Table 1. Childhood Traumatic Events Scale (CTES)

	Question 1	Question 2	Question 3	Question 4	Question 5	Question 6
CTES	Prior to the age of 17, did you experience a death of a very close friend or family member?	Prior to the age of 17, was there a major upheaval between your parents (such as divorce, separation)?	Prior to the age of 17, did you have a traumatic sexual experience (raped, molested, etc.)?	Prior to the age of 17, were you a victim of violence (child abuse, mugged or assaulted—other than sexual assault)?	Prior to the age of 17, were you extremely ill or injured?	Prior to the age of 17, did you experience any other major upheaval that you think may have shaped your life or personality significantly?

Supplemental Table 2. Example words and sentiment values for the AFINN, Bing, and NRC Dictionaries

Word	AFINN	Bing	NRC
abandon	-2	-	negative, fear, sadness
abilities	+2	-	positive
abuse	-3	negative	anger, disgust, fear, negative, sadness
absentee	-1	negative	negative, sadness
accomplish	+2	positive	joy, positive

Blank spaces indicate that the word is not present in the dictionary.

Supplemental Table 3. Pearson's correlation coefficients for sentiment scores and EPDS score

Timepoint	AFINN		BING		NRC	
	Corr	*p-value*	*Corr*	*p-value*	*Corr*	*p-value*
W2	0.14	.18	0.05	.63	0.11	.26
W6	0.09	.36	-0.01	.91	-0.03	.76
M3	0.06	.57	0.02	.86	0.02	.86
M6	0.10	.39	0.00	.99	0.08	.50
